# Poly[[aqua­[μ_2_-1,3-bis­(pyridin-4-yl)urea-κ^2^ *N* ^4^:*N* ^4′^]bis­(μ_3_-5-*tert*-butyl­isophthalato-κ^3^ *O* ^1^:*O* ^1′^:*O* ^3^)dizinc(II)] trihydrate], a double-strand coordination polymer

**DOI:** 10.1107/S2414314623006594

**Published:** 2023-08-04

**Authors:** Jason Jia, Robert L. LaDuca

**Affiliations:** a E-35 Holmes Hall, Michigan State University, Lyman Briggs College, 919 E. Shaw Lane, East Lansing, MI 48825, USA; University Koblenz-Landau, Germany

**Keywords:** crystal structure, double-strand coordination polymer, 1,3-di(pyridin-4-yl)urea, 5-*tert*-butyl­isophthalate, zinc

## Abstract

In the title compound, {[Zn_2_(C_12_H_12_O_4_)_2_(C_11_H_10_N_4_O)(H_2_O)]·3H_2_O}_*n*_, monoperiodic coordination polymer double strands are held into the triperiodic crystal structure by means of N—H⋯O hydrogen-bonding patterns between the amide groups of the 1,3-di(pyridin-4-yl)urea ligands and unligated O atoms belonging to 5-*tert*-butyl­isophthalate ligands. One of the Zn atoms displays a tetra­hedral coordination environment, while the other Zn atom adopts a five-coordinate geometry inter­mediate between square pyramidal and trigonal bipyramidal. Additionally, O—H⋯O hydrogen-bonding patterns involving the water mol­ecules of crystallization serve as a structure-stabilizing element by aggregating the double-strand motifs.

## Abstract 2

A divalent zinc one-dimensional double-strand coordination polymer, [Zn_2_(tbuip)_2_(dpu)]_*n*_, was structurally characterized by single-crystal X-ray diffraction.

## Graphical abstract

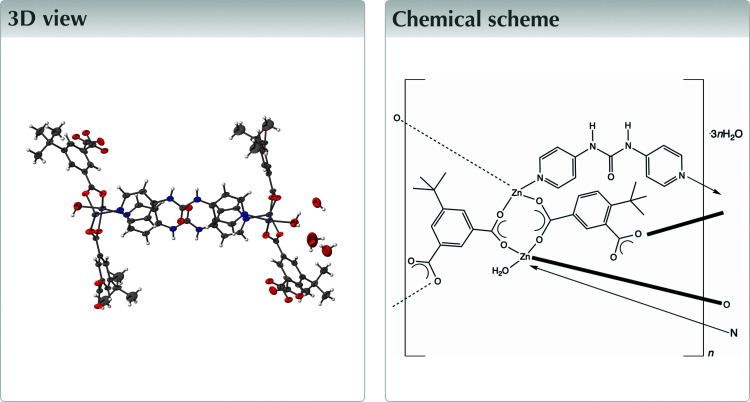

## Structure description

The title compound was isolated during an exploratory synthetic effort aiming to produce a zinc coordination polymer containing both 5-*tert*-butyl­isophthalate (tBuip) and 1,3-di(pyridin-4-yl)urea (dpu) ligands. Previously our group had isolated a zinc tBuip coord­ination polymer featuring bis­(4-pyridyl­meth­yl)piperazine coligands; this phase manifested a twofold inter­penetrated **pcu** 3-D network structure (Pochodylo & LaDuca, 2011 ▸).

**Figure 1:**
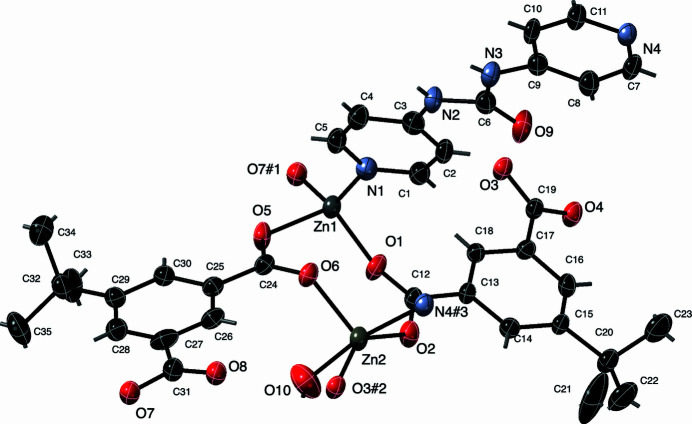
Distinct coordination environments in the title compound with full ligand set and complete {Zn_2_(OCO)_2_} dimeric cluster. Water mol­ecules of crystallization are omitted for clarity. Displacement ellipsoids are drawn at the 50% probability level. Color code: Zn, gray; O, red; N, light blue; C, black. H-atom positions are represented as sticks. Symmetry codes are as listed in Table 1 ▸.

The asymmetric unit of the title compound contains two divalent Zn atoms, two crystallographically distinct fully deprotonated tBuip ligands, one water mol­ecule bound to Zn2, one complete dpu ligand, and three water mol­ecules of crystallization. The Zn1 atoms display an [NO_3_] *pseudo*-tetra­hedral coordination environment, with a pyridyl-N donor atom from a dpu ligand, and three O atom donors belonging to three different tBuip ligands. One of the tBuip ligands has a carboxyl­ate group disordered equally in two sets of positions. In one disordered conformation, O7 binds to Zn1. In the other disordered conformation, O8*A* binds to Zn1. In contrast, the Zn2 atoms display an [NO_4_] five-coordinate environment, with a trigonality factor τ of 0.443 (Addison & Rao, 1984 ▸) indicating an inter­mediate geometry between idealized square-pyramidal and trigonal–bipyramidal forms. At Zn2, the coordination environment comprises one pyridyl-N donor atom from a bpu ligand, three O atom donors belonging to three different tBuip ligands, and a ligated water mol­ecule. Bond lengths and angles within the distinct Zn coordination environments in the title compound are listed in Table 1 ▸. Complete coordination environments and ligand sets of the asymmetric unit are shown in Fig. 1 ▸.

**Figure 2:**
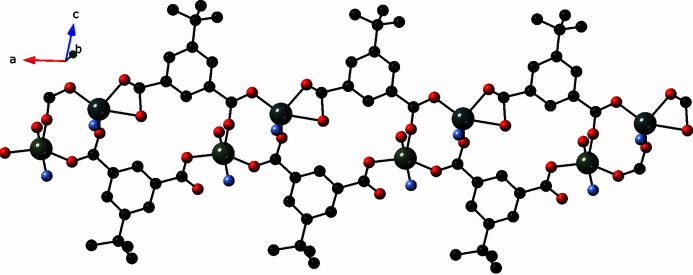
[Zn_2_(tbuip)_2_]_*n*_ coordination polymer strand in the title compound, featuring {Zn_2_(OCO)_2_} dimeric clusters.

**Figure 3:**
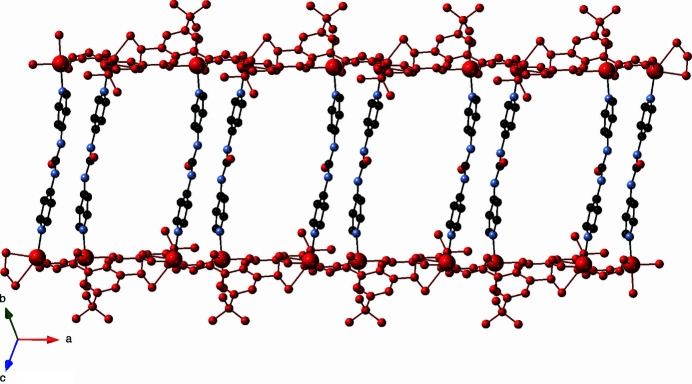
[Zn_2_(tbuip)_2_(dpu)]_*n*_ coordination polymer double strands in the title compound, with [Zn_2_(tbuip)_2_]_*n*_ strands drawn in red.

The carboxyl­ate groups of the tBuip ligands bind to Zn1 and Zn2 atoms in a *syn–anti* fashion, giving rise to bridged {Zn_2_(OCO)_2_} dimeric clusters with a Zn⋯Zn distance of 3.969 (1) Å. The full span of the tBuip ligands connect the dimeric clusters into [Zn_2_(tbuip)_2_]_*n*_ coordination polymer strands oriented along the *a* axis (Fig. 2 ▸). In turn, parallel pairs of [Zn_2_(tbuip)_2_]_*n*_ strand motifs are connected into [Zn_2_(tbuip)_2_(dpu)]_*n*_ coordination polymer double strands by tethering dpu ligands that span a Zn⋯Zn distance of 14.394 (3) Å (Fig. 3 ▸). The double strands motifs are oriented parallel to the *a* axis.

**Figure 4:**
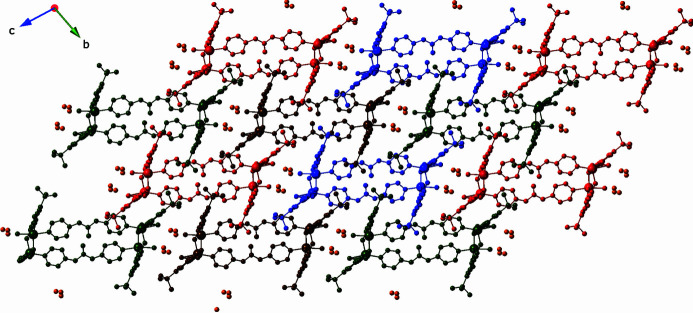
Stacking of [Zn_2_(tbuip)_2_(dpu)]_*n*_ coordination polymer double strands in the title compound, viewed down the *a* axis. The O atoms of the water mol­ecules of crystallization located between adjacent ribbons are drawn as orange spheres.

Discrete *D*(2) short water-mol­ecule chains and *C*(4) cyclic water mol­ecule tetra­mers (Infantes & Motherwell, 2002 ▸) are located between neighboring coordination polymer double strand units. These engage in O—H⋯O hydrogen-bonding patterns involving other water mol­ecules of crystallization, the water mol­ecule bound to Zn2, and unligated carboxyl­ate O atoms of the tBuip ligands. Additionally, amide groups of the dpu ligands engage in N—H⋯O hydrogen-bonding donation to unligated carboxyl­ate O atoms of the tBuip ligands. The full triperiodic crystal structure of the title compound is stabilized by these supra­molecular hydrogen-bonding inter­actions (Fig. 4 ▸). Details regarding the hydrogen-bonding patterns in the title compound are listed in Table 2 ▸.

## Synthesis and crystallization

Zn(NO_3_)_2_^.^6H_2_O (110 mg, 0.37 mmol), 5-*tert*-butyl­isophthalic acid (tBuipH_2_) (82 mg, 0.37 mmol), 1,3-di(pyridin-4-yl)urea (dpu) (79 mg, 0.37 mmol), and 0.75 ml of a 1.0 *M* NaOH solution were placed into 10 ml of distilled H_2_O in a Teflon-lined acid digestion bomb. The bomb was sealed and heated in an oven at 393 K for 48 h, and then cooled slowly to 273 K. Colorless crystals of the title complex were obtained in 55% yield.

## Refinement

Crystal data, data collection and structure refinement details are summarized in Table 3 ▸.

## Supplementary Material

Crystal structure: contains datablock(s) I, 1R. DOI: 10.1107/S2414314623006594/im4021sup1.cif

Structure factors: contains datablock(s) I. DOI: 10.1107/S2414314623006594/im4021Isup2.hkl

CCDC reference: 1959995

Additional supporting information:  crystallographic information; 3D view; checkCIF report

## Figures and Tables

**Table 1:** Selected geometric parameters (Å, °)

Zn1—O1	1.972 (4)	Zn2—O2	2.025 (3)
Zn1—O5	1.986 (3)	Zn2—O3^ii^	2.009 (3)
Zn1—O7*A* ^i^	2.074 (7)	Zn2—O6	2.017 (3)
Zn1—O8^i^	1.951 (8)	Zn2—O10	2.092 (4)
Zn1—N1	2.034 (4)	Zn2—N4^iii^	2.140 (4)
			
O1—Zn1—O5	109.03 (15)	O2—Zn2—O10	87.00 (16)
O1—Zn1—O7*A* ^i^	104.2 (2)	O2—Zn2—N4^iii^	85.75 (15)
O1—Zn1—N1	112.35 (16)	O3^ii^—Zn2—O2	142.77 (14)
O5—Zn1—O7*A* ^i^	93.7 (2)	O3^ii^—Zn2—O6	103.49 (14)
O5—Zn1—N1	105.32 (15)	O3^ii^—Zn2—O10	88.12 (15)
O8^i^—Zn1—O1	100.0 (3)	O3^ii^—Zn2—N4^iii^	93.10 (15)
O8^i^—Zn1—O5	133.3 (3)	O6—Zn2—O2	113.62 (14)
O8^i^—Zn1—O7*A* ^i^	43.0 (3)	O6—Zn2—O10	93.17 (16)
O8^i^—Zn1—N1	95.9 (3)	O6—Zn2—N4^iii^	96.64 (15)
N1—Zn1—O7*A* ^i^	129.6 (2)	O10—Zn2—N4^iii^	169.53 (17)

Symmetry codes: (i) 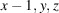 ; (ii) 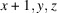 ; (iii) 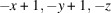 .

**Table 2:** Hydrogen-bond geometry (Å, °)

*D*—H⋯*A*	*D*—H	H⋯*A*	*D*⋯*A*	*D*—H⋯*A*
O10—H10*A*⋯O2*W*	0.89	1.88	2.658 (7)	144
O10—H10*B*⋯O1*W*	0.90	1.98	2.768 (7)	146
N3—H3⋯O4^iv^	0.88	1.92	2.761 (5)	160
O1*W*—H1*WA*⋯O1*W* ^v^	0.87	2.34	2.918 (10)	124
O2*W*—H2*WA*⋯O7^vi^	0.87	1.92	2.708 (11)	149
O2*W*—H2*WB*⋯O3*W* ^vi^	0.87	2.38	2.934 (10)	122
O3*W*—H3*WA*⋯O1^v^	0.87	2.10	2.941 (7)	164
O3*W*—H3*WB*⋯O2*W*	0.87	2.11	2.912 (11)	154

Symmetry codes: (iv) 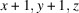 ; (v) 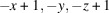 ; (vi) 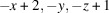 .

**Table 3:** Experimental details

Crystal data	
Chemical formula	[Zn_2_(C_12_H_12_O_4_)_2_(C_11_H_10_N_4_O)(H_2_O)]·3H_2_O
*M*_r_	857.46
Crystal system, space group	Triclinic, *P* 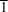
Temperature (K)	173
*a*, *b*, *c* (Å)	10.0232 (10), 10.9921 (11), 17.4698 (17)
α, β, γ (°)	100.182 (1), 100.460 (1), 101.335 (1)
*V* (Å^3^)	1810.2 (3)
*Z*	2
Radiation type	Mo *K*α
μ (mm^−1^)	1.40
Crystal size (mm)	0.13 × 0.11 × 0.10

Data collection	
Diffractometer	Bruker APEXII CCD
Absorption correction	Multi-scan (*SADABS*; Krause *et al.*, 2015 ▸)
*T*_min_, *T*_max_	0.696, 0.745
No. of measured, independent and observed [*I* > 2σ(*I*)] reflections	25998, 6575, 4380
*R*_int_	0.077
(sin θ/λ)_max_ (Å^−1^)	0.602

Refinement	
*R*[*F* ^2^ > 2σ(*F* ^2^)], *wR*(*F* ^2^), *S*	0.057, 0.155, 1.03
No. of reflections	6575
No. of parameters	493
No. of restraints	5
H-atom treatment	H-atom parameters constrained
Δρ_max_, Δρ_min_ (e Å^−3^)	1.33, −0.65

Computer programs: *COSMO* (Bruker, 2009 ▸), *SAINT* (Bruker, 2014 ▸), *SHELXT* (Sheldrick, 2015*a* ▸), *SHELXL* (Sheldrick, 2015*b* ▸), *CrystalMaker X* (Palmer, 2020 ▸), and *OLEX2* (Dolomanov *et al.*, 2009 ▸).

## full crystallographic data

### Crystal data

**Table:**

[Zn_2_(C_12_H_12_O_4_)_2_(C_11_H_10_N_4_O)(H_2_O)]·3H_2_O	*Z* = 2
*M**_r_* = 857.46	*F*(000) = 888
Triclinic, *P*1	*D*_x_ = 1.573 Mg m^−^^3^
*a* = 10.0232 (10) Å	Mo *K*α radiation, λ = 0.71073 Å
*b* = 10.9921 (11) Å	Cell parameters from 5602 reflections
*c* = 17.4698 (17) Å	θ = 2.4–25.3°
α = 100.182 (1)°	µ = 1.40 mm^−^^1^
β = 100.460 (1)°	*T* = 173 K
γ = 101.335 (1)°	Chunk, colourless
*V* = 1810.2 (3) Å^3^	0.13 × 0.11 × 0.10 mm

### Data collection

**Table:**

Bruker APEXII CCD diffractometer	6575 independent reflections
Radiation source: sealed tube	4380 reflections with *I* > 2σ(*I*)
Graphite monochromator	*R*_int_ = 0.077
Detector resolution: 8.36 pixels mm^-1^	θ_max_ = 25.3°, θ_min_ = 1.2°
ω scans	*h* = −12→12
Absorption correction: multi-scan (SADABS; Krause *et al.*, 2015)	*k* = −13→13
*T*_min_ = 0.696, *T*_max_ = 0.745	*l* = −20→21
25998 measured reflections	

### Refinement

**Table:**

Refinement on *F*^2^	Primary atom site location: dual
Least-squares matrix: full	Hydrogen site location: mixed
*R*[*F*^2^ > 2σ(*F*^2^)] = 0.057	H-atom parameters constrained
*wR*(*F*^2^) = 0.155	*w* = 1/[σ^2^(*F*_o_^2^) + (0.0721*P*)^2^ + 1.4791*P*] where *P* = (*F*_o_^2^ + 2*F*_c_^2^)/3
*S* = 1.03	(Δ/σ)_max_ < 0.001
6575 reflections	Δρ_max_ = 1.33 e Å^−^^3^
493 parameters	Δρ_min_ = −0.65 e Å^−^^3^
5 restraints	

### Special details

**Table:**

Experimental. Data were collected using a BRUKER CCD (charge coupled device) based diffractometer equipped with an Oxford low-temperature apparatus operating at 173 K. A suitable crystal was chosen and mounted on a nylon loop using Paratone oil. Data were measured using omega scans of 0.5° per frame for 30 s. The total number of images were based on results from the program COSMO where redundancy was expected to be 4 and completeness to 0.83Å to 100%. Cell parameters were retrieved using APEX II software and refined using SAINT on all observed reflections.Data reduction was performed using the SAINT software which corrects for Lp. Scaling and absorption corrections were applied using SADABS6 multi-scan technique, supplied by George Sheldrick. The structure was solved by the direct method using the SHELXT program and refined by least squares method on F2, SHELXL, incorporated in OLEX2.
Geometry. All esds (except the esd in the dihedral angle between two l.s. planes) are estimated using the full covariance matrix. The cell esds are taken into account individually in the estimation of esds in distances, angles and torsion angles; correlations between esds in cell parameters are only used when they are defined by crystal symmetry. An approximate (isotropic) treatment of cell esds is used for estimating esds involving l.s. planes.
Refinement. The structure was refined by Least Squares using version 2018/3 of XL (Sheldrick, 2015) incorporated in Olex2 (Dolomanov *et al.*, 2009). All non-hydrogen atoms were refined anisotropically. All H atoms were placed in calculated positions with C—H = 0.98 Å for CH_3_ groups and 0.95 Å for phenyl and pyridyl groups, N—H = 0.88 Å, 0.88 Å for the coordinated water ligand and 0.87 Å for solvent water molecules. Hydrogen atoms were constrained to ride on their parent atoms, with *U*_iso_(H) = 1.2 *U*_eq_(C,*N*) for aromatic hydrogen atoms and N—H groups and with *U*_iso_(H) = 1.2 *U*_eq_(C,*O*) for methyl groups and all oxygen atoms.

### Fractional atomic coordinates and isotropic or equivalent isotropic displacement parameters (Å^2^)

**Table:**

	*x*	*y*	*z*	*U*_iso_*/*U*_eq_	Occ. (<1)
Zn1	0.38132 (6)	0.30660 (6)	0.39727 (3)	0.02965 (19)	
Zn2	0.61061 (6)	0.11823 (6)	0.28134 (3)	0.03045 (19)	
O1	0.3106 (4)	0.1265 (3)	0.3413 (2)	0.0403 (9)	
O2	0.4073 (3)	0.0235 (3)	0.2530 (2)	0.0359 (9)	
O3	−0.1993 (3)	0.1023 (3)	0.26575 (19)	0.0326 (8)	
O4	−0.3317 (3)	−0.0717 (3)	0.1824 (2)	0.0341 (8)	
O5	0.5679 (3)	0.3284 (3)	0.46735 (18)	0.0330 (8)	
O6	0.6496 (4)	0.2880 (3)	0.35802 (19)	0.0355 (9)	
O7	1.2637 (7)	0.2507 (7)	0.5132 (4)	0.0328 (10)	0.5
O7A	1.2822 (8)	0.3242 (7)	0.4917 (4)	0.0328 (10)	0.5
O8	1.2077 (9)	0.3474 (8)	0.4165 (5)	0.039 (2)	0.5
O8A	1.1426 (8)	0.3094 (8)	0.3755 (5)	0.0328 (10)	0.5
O9	0.3808 (4)	0.5378 (4)	0.0421 (2)	0.0488 (11)	
O10	0.6375 (4)	0.0199 (4)	0.3730 (3)	0.0614 (12)	
H10A	0.695720	−0.030452	0.364270	0.092*	
H10B	0.558890	−0.036272	0.372250	0.092*	
N1	0.4125 (4)	0.4238 (4)	0.3209 (2)	0.0308 (10)	
N2	0.5062 (4)	0.6579 (4)	0.1645 (2)	0.0343 (10)	
H2	0.564503	0.731560	0.187950	0.041*	
N3	0.5180 (4)	0.7370 (4)	0.0532 (2)	0.0351 (11)	
H3	0.581303	0.798638	0.087919	0.042*	
N4	0.4375 (4)	0.8144 (4)	−0.1743 (2)	0.0309 (10)	
C1	0.3507 (5)	0.3873 (5)	0.2435 (3)	0.0341 (12)	
H1	0.285912	0.306966	0.225244	0.041*	
C2	0.3760 (5)	0.4602 (5)	0.1885 (3)	0.0341 (12)	
H2A	0.329037	0.430044	0.133989	0.041*	
C3	0.4702 (5)	0.5774 (5)	0.2135 (3)	0.0328 (12)	
C4	0.5331 (5)	0.6168 (5)	0.2951 (3)	0.0349 (13)	
H4	0.596677	0.697517	0.315202	0.042*	
C5	0.5028 (5)	0.5386 (5)	0.3456 (3)	0.0338 (12)	
H5	0.547361	0.566630	0.400565	0.041*	
C6	0.4606 (5)	0.6357 (5)	0.0825 (3)	0.0332 (12)	
C7	0.3781 (5)	0.7017 (5)	−0.1630 (3)	0.0359 (13)	
H7	0.316776	0.641538	−0.207380	0.043*	
C8	0.4006 (6)	0.6666 (5)	−0.0894 (3)	0.0377 (13)	
H8	0.356602	0.584512	−0.084316	0.045*	
C9	0.4883 (5)	0.7540 (5)	−0.0244 (3)	0.0319 (12)	
C10	0.5527 (5)	0.8704 (5)	−0.0363 (3)	0.0338 (12)	
H10	0.616375	0.931550	0.006766	0.041*	
C11	0.5243 (5)	0.8970 (5)	−0.1101 (3)	0.0352 (13)	
H11	0.568231	0.978228	−0.116561	0.042*	
C12	0.3050 (5)	0.0536 (5)	0.2759 (3)	0.0270 (11)	
C13	0.1629 (5)	−0.0044 (4)	0.2218 (3)	0.0248 (11)	
C14	0.1506 (5)	−0.0888 (5)	0.1498 (3)	0.0280 (11)	
H14	0.232221	−0.110072	0.136486	0.034*	
C15	0.0222 (5)	−0.1428 (4)	0.0969 (3)	0.0257 (11)	
C16	−0.0950 (5)	−0.1130 (4)	0.1203 (3)	0.0265 (11)	
H16	−0.184452	−0.152363	0.086602	0.032*	
C17	−0.0862 (5)	−0.0276 (4)	0.1912 (3)	0.0255 (11)	
C18	0.0447 (5)	0.0293 (4)	0.2418 (3)	0.0255 (11)	
H18	0.052938	0.090366	0.289325	0.031*	
C19	−0.2145 (5)	0.0018 (5)	0.2143 (3)	0.0256 (11)	
C20	0.0077 (5)	−0.2321 (5)	0.0162 (3)	0.0375 (13)	
C21	0.1438 (8)	−0.2552 (7)	0.0016 (4)	0.0754 (13)	
H21A	0.200943	−0.177036	−0.006137	0.113*	
H21B	0.126774	−0.322976	−0.046180	0.113*	
H21C	0.193123	−0.280747	0.047701	0.113*	
C22	−0.0783 (8)	−0.3659 (7)	0.0180 (4)	0.0754 (13)	
H22A	−0.025505	−0.400700	0.058277	0.113*	
H22B	−0.095853	−0.422886	−0.034495	0.113*	
H22C	−0.167438	−0.358048	0.031455	0.113*	
C23	−0.0777 (8)	−0.1923 (7)	−0.0508 (4)	0.0754 (13)	
H23A	−0.170808	−0.192197	−0.040841	0.113*	
H23B	−0.086323	−0.252084	−0.101222	0.113*	
H23C	−0.031702	−0.106620	−0.054091	0.113*	
C24	0.6671 (5)	0.3144 (4)	0.4332 (3)	0.0273 (11)	
C25	0.8089 (5)	0.3317 (4)	0.4840 (3)	0.0272 (11)	
C26	0.9219 (5)	0.3176 (4)	0.4499 (3)	0.0298 (12)	
H26	0.910061	0.299218	0.393564	0.036*	
C27	1.0512 (5)	0.3304 (5)	0.4988 (3)	0.0358 (13)	
C28	1.0671 (5)	0.3553 (5)	0.5811 (3)	0.0343 (12)	
H28	1.155945	0.361394	0.613817	0.041*	
C29	0.9571 (5)	0.3715 (5)	0.6173 (3)	0.0279 (11)	
C30	0.8288 (5)	0.3610 (4)	0.5669 (3)	0.0261 (11)	
H30	0.752307	0.374166	0.589668	0.031*	
C31	1.1850 (15)	0.3084 (12)	0.4767 (7)	0.0328 (10)	0.5
C31A	1.1618 (14)	0.3207 (14)	0.4492 (8)	0.0328 (10)	0.5
C32	0.9775 (6)	0.4005 (5)	0.7085 (3)	0.0369 (13)	
C33	0.8481 (6)	0.3313 (6)	0.7321 (3)	0.0513 (16)	
H33A	0.767100	0.362334	0.710670	0.077*	
H33B	0.863868	0.347516	0.790444	0.077*	
H33C	0.830626	0.239633	0.710421	0.077*	
C34	0.9951 (7)	0.5441 (6)	0.7375 (4)	0.0598 (18)	
H34A	1.079881	0.590080	0.725212	0.090*	
H34B	1.002733	0.564110	0.795337	0.090*	
H34C	0.913940	0.569712	0.710635	0.090*	
C35	1.1038 (7)	0.3611 (7)	0.7494 (4)	0.0589 (18)	
H35A	1.091752	0.269102	0.731535	0.088*	
H35B	1.113470	0.382567	0.807333	0.088*	
H35C	1.187780	0.405971	0.735757	0.088*	
O2W	0.8558 (8)	−0.0788 (8)	0.4110 (5)	0.131 (3)	
H2WA	0.849857	−0.131613	0.442563	0.196*	
H2WB	0.928881	−0.018318	0.435648	0.196*	
O3W	0.8410 (7)	0.0382 (6)	0.5714 (4)	0.104 (2)	
H3WA	0.790799	−0.021227	0.588147	0.156*	
H3WB	0.820815	0.012124	0.519791	0.156*	
O1W	0.4387 (6)	−0.1147 (5)	0.4373 (3)	0.0763 (15)	
H1WA	0.410888	−0.061226	0.470248	0.114*	
H1WB	0.509999	−0.131385	0.466540	0.114*	

### Atomic displacement parameters (Å^2^)

**Table:**

	*U*^11^	*U*^22^	*U*^33^	*U*^12^	*U*^13^	*U*^23^
Zn1	0.0193 (3)	0.0393 (4)	0.0255 (3)	0.0067 (3)	0.0028 (2)	−0.0030 (3)
Zn2	0.0175 (3)	0.0445 (4)	0.0246 (3)	0.0073 (3)	0.0027 (2)	−0.0023 (3)
O1	0.031 (2)	0.044 (2)	0.033 (2)	0.0044 (17)	0.0022 (17)	−0.0139 (17)
O2	0.0170 (19)	0.053 (2)	0.031 (2)	0.0054 (17)	0.0032 (15)	−0.0033 (17)
O3	0.0206 (19)	0.041 (2)	0.0295 (19)	0.0087 (16)	0.0035 (15)	−0.0086 (16)
O4	0.0195 (19)	0.041 (2)	0.032 (2)	0.0019 (16)	0.0032 (16)	−0.0066 (16)
O5	0.0188 (18)	0.057 (2)	0.0183 (17)	0.0039 (17)	0.0055 (14)	0.0003 (16)
O6	0.044 (2)	0.038 (2)	0.0202 (18)	0.0099 (18)	0.0049 (16)	−0.0021 (15)
O7	0.020 (2)	0.045 (3)	0.031 (2)	0.009 (2)	0.0063 (19)	−0.001 (2)
O7A	0.020 (2)	0.045 (3)	0.031 (2)	0.009 (2)	0.0063 (19)	−0.001 (2)
O8	0.041 (6)	0.040 (5)	0.049 (5)	0.015 (4)	0.031 (4)	0.010 (4)
O8A	0.020 (2)	0.045 (3)	0.031 (2)	0.009 (2)	0.0063 (19)	−0.001 (2)
O9	0.054 (3)	0.047 (2)	0.028 (2)	−0.009 (2)	−0.0043 (19)	0.0015 (18)
O10	0.035 (2)	0.086 (3)	0.072 (3)	0.018 (2)	0.010 (2)	0.037 (3)
N1	0.021 (2)	0.039 (3)	0.031 (2)	0.006 (2)	0.0085 (19)	0.002 (2)
N2	0.032 (3)	0.035 (2)	0.026 (2)	−0.003 (2)	−0.0008 (19)	0.0005 (19)
N3	0.032 (3)	0.037 (3)	0.028 (2)	0.000 (2)	0.001 (2)	−0.001 (2)
N4	0.024 (2)	0.039 (3)	0.026 (2)	0.007 (2)	0.0038 (19)	−0.0009 (19)
C1	0.019 (3)	0.039 (3)	0.037 (3)	0.005 (2)	−0.001 (2)	−0.002 (2)
C2	0.027 (3)	0.041 (3)	0.026 (3)	0.006 (2)	−0.002 (2)	−0.003 (2)
C3	0.021 (3)	0.041 (3)	0.036 (3)	0.007 (2)	0.007 (2)	0.006 (2)
C4	0.023 (3)	0.042 (3)	0.033 (3)	0.002 (2)	0.003 (2)	−0.001 (2)
C5	0.024 (3)	0.043 (3)	0.028 (3)	0.004 (2)	0.002 (2)	−0.001 (2)
C6	0.029 (3)	0.036 (3)	0.031 (3)	0.006 (3)	0.003 (2)	0.004 (2)
C7	0.026 (3)	0.040 (3)	0.030 (3)	0.002 (2)	0.001 (2)	−0.009 (2)
C8	0.029 (3)	0.045 (3)	0.032 (3)	0.004 (3)	0.001 (2)	0.002 (3)
C9	0.025 (3)	0.043 (3)	0.027 (3)	0.012 (2)	0.005 (2)	0.001 (2)
C10	0.028 (3)	0.043 (3)	0.021 (3)	0.005 (2)	−0.002 (2)	−0.007 (2)
C11	0.030 (3)	0.040 (3)	0.027 (3)	0.001 (2)	0.001 (2)	−0.001 (2)
C12	0.019 (3)	0.033 (3)	0.024 (3)	0.004 (2)	0.003 (2)	0.001 (2)
C13	0.025 (3)	0.027 (3)	0.019 (2)	0.003 (2)	0.003 (2)	0.001 (2)
C14	0.021 (3)	0.033 (3)	0.029 (3)	0.008 (2)	0.005 (2)	0.002 (2)
C15	0.023 (3)	0.028 (3)	0.022 (2)	0.003 (2)	0.004 (2)	−0.001 (2)
C16	0.013 (2)	0.034 (3)	0.027 (3)	0.000 (2)	0.001 (2)	0.002 (2)
C17	0.026 (3)	0.026 (3)	0.023 (3)	0.007 (2)	0.006 (2)	0.002 (2)
C18	0.021 (3)	0.027 (3)	0.026 (3)	0.004 (2)	0.002 (2)	0.002 (2)
C19	0.022 (3)	0.029 (3)	0.022 (3)	0.004 (2)	0.002 (2)	0.000 (2)
C20	0.019 (3)	0.045 (3)	0.038 (3)	0.009 (2)	0.002 (2)	−0.013 (3)
C21	0.089 (3)	0.075 (3)	0.048 (2)	0.023 (3)	0.009 (2)	−0.020 (2)
C22	0.089 (3)	0.075 (3)	0.048 (2)	0.023 (3)	0.009 (2)	−0.020 (2)
C23	0.089 (3)	0.075 (3)	0.048 (2)	0.023 (3)	0.009 (2)	−0.020 (2)
C24	0.029 (3)	0.024 (3)	0.026 (3)	0.002 (2)	0.007 (2)	0.003 (2)
C25	0.030 (3)	0.024 (3)	0.027 (3)	0.005 (2)	0.009 (2)	0.004 (2)
C26	0.032 (3)	0.026 (3)	0.031 (3)	0.003 (2)	0.016 (2)	0.001 (2)
C27	0.027 (3)	0.022 (3)	0.055 (4)	−0.002 (2)	0.019 (3)	−0.002 (2)
C28	0.022 (3)	0.035 (3)	0.040 (3)	0.004 (2)	0.002 (2)	0.001 (2)
C29	0.019 (3)	0.031 (3)	0.033 (3)	0.007 (2)	0.005 (2)	0.006 (2)
C30	0.022 (3)	0.031 (3)	0.027 (3)	0.006 (2)	0.010 (2)	0.005 (2)
C31	0.020 (2)	0.045 (3)	0.031 (2)	0.009 (2)	0.0063 (19)	−0.001 (2)
C31A	0.020 (2)	0.045 (3)	0.031 (2)	0.009 (2)	0.0063 (19)	−0.001 (2)
C32	0.031 (3)	0.048 (3)	0.031 (3)	0.013 (3)	0.001 (2)	0.007 (2)
C33	0.043 (4)	0.074 (5)	0.032 (3)	0.007 (3)	0.004 (3)	0.013 (3)
C34	0.063 (5)	0.060 (4)	0.044 (4)	0.015 (4)	−0.002 (3)	−0.007 (3)
C35	0.043 (4)	0.094 (5)	0.045 (4)	0.029 (4)	0.000 (3)	0.022 (4)
O2W	0.111 (6)	0.140 (6)	0.190 (7)	0.071 (5)	0.044 (5)	0.104 (6)
O3W	0.113 (5)	0.104 (5)	0.094 (4)	0.004 (4)	0.025 (4)	0.038 (4)
O1W	0.095 (4)	0.068 (3)	0.065 (3)	0.004 (3)	0.023 (3)	0.021 (3)

### Geometric parameters (Å, º)

**Table:**

Zn1—O1	1.972 (4)	C14—H14	0.9500
Zn1—O5	1.986 (3)	C14—C15	1.392 (6)
Zn1—O7A^i^	2.074 (7)	C15—C16	1.390 (6)
Zn1—O8^i^	1.951 (8)	C15—C20	1.533 (7)
Zn1—O8A^i^	2.361 (8)	C16—H16	0.9500
Zn1—N1	2.034 (4)	C16—C17	1.394 (6)
Zn2—O2	2.025 (3)	C17—C18	1.398 (7)
Zn2—O3^ii^	2.009 (3)	C17—C19	1.491 (7)
Zn2—O6	2.017 (3)	C18—H18	0.9500
Zn2—O10	2.092 (4)	C20—C21	1.494 (9)
Zn2—N4^iii^	2.140 (4)	C20—C22	1.561 (9)
O1—C12	1.259 (6)	C20—C23	1.503 (9)
O2—C12	1.247 (6)	C21—H21A	0.9800
O3—C19	1.259 (5)	C21—H21B	0.9800
O4—C19	1.254 (6)	C21—H21C	0.9800
O5—C24	1.270 (6)	C22—H22A	0.9800
O6—C24	1.265 (5)	C22—H22B	0.9800
O7—C31	1.259 (14)	C22—H22C	0.9800
O7A—C31A	1.287 (14)	C23—H23A	0.9800
O8—C31	1.246 (14)	C23—H23B	0.9800
O8A—C31A	1.246 (15)	C23—H23C	0.9800
O9—C6	1.218 (6)	C24—C25	1.490 (7)
O10—H10A	0.8944	C25—C26	1.394 (7)
O10—H10B	0.8969	C25—C30	1.393 (6)
N1—C1	1.334 (6)	C26—H26	0.9500
N1—C5	1.347 (6)	C26—C27	1.383 (7)
N2—H2	0.8800	C27—C28	1.389 (7)
N2—C3	1.379 (7)	C27—C31	1.512 (15)
N2—C6	1.384 (6)	C27—C31A	1.534 (16)
N3—H3	0.8800	C28—H28	0.9500
N3—C6	1.374 (7)	C28—C29	1.391 (7)
N3—C9	1.388 (6)	C29—C30	1.392 (6)
N4—C7	1.330 (7)	C29—C32	1.537 (7)
N4—C11	1.347 (6)	C30—H30	0.9500
C1—H1	0.9500	C32—C33	1.533 (8)
C1—C2	1.382 (7)	C32—C34	1.534 (8)
C2—H2A	0.9500	C32—C35	1.516 (7)
C2—C3	1.384 (7)	C33—H33A	0.9800
C3—C4	1.402 (7)	C33—H33B	0.9800
C4—H4	0.9500	C33—H33C	0.9800
C4—C5	1.369 (7)	C34—H34A	0.9800
C5—H5	0.9500	C34—H34B	0.9800
C7—H7	0.9500	C34—H34C	0.9800
C7—C8	1.398 (7)	C35—H35A	0.9800
C8—H8	0.9500	C35—H35B	0.9800
C8—C9	1.381 (7)	C35—H35C	0.9800
C9—C10	1.384 (7)	O2W—H2WA	0.8697
C10—H10	0.9500	O2W—H2WB	0.8701
C10—C11	1.365 (7)	O3W—H3WA	0.8700
C11—H11	0.9500	O3W—H3WB	0.8699
C12—C13	1.507 (6)	O1W—H1WA	0.8698
C13—C14	1.395 (6)	O1W—H1WB	0.8702
C13—C18	1.392 (6)		
			
O1—Zn1—O5	109.03 (15)	C16—C15—C20	120.5 (4)
O1—Zn1—O7A^i^	104.2 (2)	C15—C16—H16	118.8
O1—Zn1—O8A^i^	84.1 (2)	C15—C16—C17	122.5 (4)
O1—Zn1—N1	112.35 (16)	C17—C16—H16	118.8
O1—Zn1—C31A^i^	94.5 (3)	C16—C17—C18	119.2 (4)
O5—Zn1—O7A^i^	93.7 (2)	C16—C17—C19	120.8 (4)
O5—Zn1—O8A^i^	152.6 (2)	C18—C17—C19	119.9 (4)
O5—Zn1—N1	105.32 (15)	C13—C18—C17	119.3 (4)
O5—Zn1—C31A^i^	123.8 (3)	C13—C18—H18	120.3
O7A^i^—Zn1—C31A^i^	30.1 (3)	C17—C18—H18	120.3
O8^i^—Zn1—O1	100.0 (3)	O3—C19—C17	117.3 (4)
O8^i^—Zn1—O5	133.3 (3)	O4—C19—O3	122.5 (4)
O8^i^—Zn1—O7A^i^	43.0 (3)	O4—C19—C17	120.2 (4)
O8^i^—Zn1—O8A^i^	20.1 (3)	C15—C20—C22	107.9 (5)
O8^i^—Zn1—N1	95.9 (3)	C21—C20—C15	113.2 (4)
O8^i^—Zn1—C31A^i^	15.7 (4)	C21—C20—C22	104.2 (5)
O8A^i^—Zn1—C31A^i^	29.1 (3)	C21—C20—C23	114.3 (6)
N1—Zn1—O7A^i^	129.6 (2)	C23—C20—C15	111.6 (4)
N1—Zn1—O8A^i^	90.7 (2)	C23—C20—C22	104.9 (5)
N1—Zn1—C31A^i^	111.5 (3)	C20—C21—H21A	109.5
O2—Zn2—O10	87.00 (16)	C20—C21—H21B	109.5
O2—Zn2—N4^iii^	85.75 (15)	C20—C21—H21C	109.5
O3^ii^—Zn2—O2	142.77 (14)	H21A—C21—H21B	109.5
O3^ii^—Zn2—O6	103.49 (14)	H21A—C21—H21C	109.5
O3^ii^—Zn2—O10	88.12 (15)	H21B—C21—H21C	109.5
O3^ii^—Zn2—N4^iii^	93.10 (15)	C20—C22—H22A	109.5
O6—Zn2—O2	113.62 (14)	C20—C22—H22B	109.5
O6—Zn2—O10	93.17 (16)	C20—C22—H22C	109.5
O6—Zn2—N4^iii^	96.64 (15)	H22A—C22—H22B	109.5
O10—Zn2—N4^iii^	169.53 (17)	H22A—C22—H22C	109.5
C12—O1—Zn1	140.4 (4)	H22B—C22—H22C	109.5
C12—O2—Zn2	130.1 (3)	C20—C23—H23A	109.5
C19—O3—Zn2^i^	108.0 (3)	C20—C23—H23B	109.5
C24—O5—Zn1	116.9 (3)	C20—C23—H23C	109.5
C24—O6—Zn2	130.1 (3)	H23A—C23—H23B	109.5
C31—O8—Zn1^ii^	107.2 (8)	H23A—C23—H23C	109.5
Zn2—O10—H10A	109.6	H23B—C23—H23C	109.5
Zn2—O10—H10B	111.9	O5—C24—C25	118.3 (4)
H10A—O10—H10B	102.2	O6—C24—O5	122.3 (5)
C1—N1—Zn1	121.4 (4)	O6—C24—C25	119.5 (4)
C1—N1—C5	117.2 (4)	C26—C25—C24	120.9 (4)
C5—N1—Zn1	121.2 (3)	C30—C25—C24	119.7 (4)
C3—N2—H2	116.4	C30—C25—C26	119.4 (5)
C3—N2—C6	127.1 (4)	C25—C26—H26	120.2
C6—N2—H2	116.4	C27—C26—C25	119.5 (5)
C6—N3—H3	116.1	C27—C26—H26	120.2
C6—N3—C9	127.9 (4)	C26—C27—C28	119.9 (5)
C9—N3—H3	116.1	C26—C27—C31	129.5 (6)
C7—N4—Zn2^iii^	125.5 (3)	C26—C27—C31A	110.7 (6)
C7—N4—C11	116.5 (4)	C28—C27—C31	110.3 (6)
C11—N4—Zn2^iii^	117.7 (4)	C28—C27—C31A	129.3 (6)
N1—C1—H1	118.3	C27—C28—H28	118.9
N1—C1—C2	123.4 (5)	C27—C28—C29	122.2 (5)
C2—C1—H1	118.3	C29—C28—H28	118.9
C1—C2—H2A	120.3	C28—C29—C30	116.8 (5)
C1—C2—C3	119.5 (5)	C28—C29—C32	121.3 (4)
C3—C2—H2A	120.3	C30—C29—C32	121.9 (4)
N2—C3—C2	125.1 (5)	C25—C30—H30	118.9
N2—C3—C4	117.8 (5)	C29—C30—C25	122.2 (4)
C2—C3—C4	117.1 (5)	C29—C30—H30	118.9
C3—C4—H4	120.1	O7—C31—C27	123.7 (10)
C5—C4—C3	119.8 (5)	O8—C31—O7	122.4 (12)
C5—C4—H4	120.1	O8—C31—C27	113.8 (10)
N1—C5—C4	123.0 (5)	O7A—C31A—C27	112.9 (10)
N1—C5—H5	118.5	O8A—C31A—O7A	121.1 (13)
C4—C5—H5	118.5	O8A—C31A—C27	126.0 (10)
O9—C6—N2	123.6 (5)	C27—C31A—Zn1^ii^	166.9 (7)
O9—C6—N3	125.0 (5)	C33—C32—C29	110.2 (4)
N3—C6—N2	111.3 (4)	C33—C32—C34	108.2 (5)
N4—C7—H7	118.1	C34—C32—C29	108.1 (4)
N4—C7—C8	123.8 (5)	C35—C32—C29	112.3 (4)
C8—C7—H7	118.1	C35—C32—C33	108.5 (5)
C7—C8—H8	120.8	C35—C32—C34	109.5 (5)
C9—C8—C7	118.4 (5)	C32—C33—H33A	109.5
C9—C8—H8	120.8	C32—C33—H33B	109.5
C8—C9—N3	126.1 (5)	C32—C33—H33C	109.5
C8—C9—C10	118.0 (5)	H33A—C33—H33B	109.5
C10—C9—N3	115.9 (4)	H33A—C33—H33C	109.5
C9—C10—H10	120.2	H33B—C33—H33C	109.5
C11—C10—C9	119.7 (5)	C32—C34—H34A	109.5
C11—C10—H10	120.2	C32—C34—H34B	109.5
N4—C11—C10	123.6 (5)	C32—C34—H34C	109.5
N4—C11—H11	118.2	H34A—C34—H34B	109.5
C10—C11—H11	118.2	H34A—C34—H34C	109.5
O1—C12—C13	117.3 (4)	H34B—C34—H34C	109.5
O2—C12—O1	125.3 (4)	C32—C35—H35A	109.5
O2—C12—C13	117.4 (4)	C32—C35—H35B	109.5
C14—C13—C12	119.5 (4)	C32—C35—H35C	109.5
C18—C13—C12	120.5 (4)	H35A—C35—H35B	109.5
C18—C13—C14	119.9 (4)	H35A—C35—H35C	109.5
C13—C14—H14	119.1	H35B—C35—H35C	109.5
C15—C14—C13	121.8 (4)	H2WA—O2W—H2WB	104.5
C15—C14—H14	119.1	H3WA—O3W—H3WB	104.5
C14—C15—C20	122.4 (4)	H1WA—O1W—H1WB	104.5
C16—C15—C14	117.1 (4)		
			
Zn1—O1—C12—O2	67.4 (8)	C12—C13—C14—C15	−178.5 (4)
Zn1—O1—C12—C13	−114.1 (5)	C12—C13—C18—C17	−178.9 (4)
Zn1—O5—C24—O6	0.1 (6)	C13—C14—C15—C16	−2.5 (7)
Zn1—O5—C24—C25	179.2 (3)	C13—C14—C15—C20	177.8 (5)
Zn1^ii^—O7A—C31A—O8A	0.1 (14)	C14—C13—C18—C17	3.5 (7)
Zn1^ii^—O7A—C31A—C27	179.5 (8)	C14—C15—C16—C17	3.5 (7)
Zn1^ii^—O8—C31—O7	−1.9 (15)	C14—C15—C20—C21	0.7 (8)
Zn1^ii^—O8—C31—C27	−178.7 (7)	C14—C15—C20—C22	115.4 (6)
Zn1^ii^—O8A—C31A—O7A	−0.1 (12)	C14—C15—C20—C23	−129.9 (6)
Zn1^ii^—O8A—C31A—C27	−179.4 (13)	C15—C16—C17—C18	−1.0 (7)
Zn1—N1—C1—C2	−175.5 (4)	C15—C16—C17—C19	−179.9 (4)
Zn1—N1—C5—C4	175.8 (4)	C16—C15—C20—C21	−179.0 (5)
Zn2—O2—C12—O1	−26.6 (8)	C16—C15—C20—C22	−64.3 (6)
Zn2—O2—C12—C13	155.0 (3)	C16—C15—C20—C23	50.4 (7)
Zn2^i^—O3—C19—O4	−1.4 (6)	C16—C17—C18—C13	−2.6 (7)
Zn2^i^—O3—C19—C17	178.2 (3)	C16—C17—C19—O3	−161.0 (4)
Zn2—O6—C24—O5	−94.7 (5)	C16—C17—C19—O4	18.6 (7)
Zn2—O6—C24—C25	86.2 (5)	C18—C13—C14—C15	−0.9 (7)
Zn2^iii^—N4—C7—C8	−172.4 (4)	C18—C17—C19—O3	20.1 (7)
Zn2^iii^—N4—C11—C10	173.3 (4)	C18—C17—C19—O4	−160.3 (4)
O1—C12—C13—C14	−178.1 (5)	C19—C17—C18—C13	176.4 (4)
O1—C12—C13—C18	4.4 (7)	C20—C15—C16—C17	−176.8 (5)
O2—C12—C13—C14	0.5 (7)	C24—C25—C26—C27	−178.0 (4)
O2—C12—C13—C18	−177.1 (4)	C24—C25—C30—C29	176.5 (4)
O5—C24—C25—C26	180.0 (4)	C25—C26—C27—C28	1.0 (7)
O5—C24—C25—C30	0.8 (7)	C25—C26—C27—C31	174.0 (8)
O6—C24—C25—C26	−0.9 (7)	C25—C26—C27—C31A	−175.9 (7)
O6—C24—C25—C30	179.9 (4)	C26—C25—C30—C29	−2.7 (7)
N1—C1—C2—C3	0.2 (8)	C26—C27—C28—C29	−1.8 (8)
N2—C3—C4—C5	−178.6 (5)	C26—C27—C31—O7	−136.4 (10)
N3—C9—C10—C11	−177.1 (5)	C26—C27—C31—O8	40.4 (14)
N4—C7—C8—C9	0.7 (8)	C26—C27—C31A—Zn1^ii^	−174 (4)
C1—N1—C5—C4	−0.4 (7)	C26—C27—C31A—O7A	−175.9 (9)
C1—C2—C3—N2	178.9 (5)	C26—C27—C31A—O8A	3.5 (16)
C1—C2—C3—C4	−1.4 (7)	C27—C28—C29—C30	0.3 (7)
C2—C3—C4—C5	1.6 (7)	C27—C28—C29—C32	−179.2 (5)
C3—N2—C6—O9	−0.7 (9)	C28—C27—C31—O7	37.2 (14)
C3—N2—C6—N3	−179.9 (5)	C28—C27—C31—O8	−146.0 (9)
C3—C4—C5—N1	−0.7 (8)	C28—C27—C31A—Zn1^ii^	9 (4)
C5—N1—C1—C2	0.7 (7)	C28—C27—C31A—O7A	7.5 (15)
C6—N2—C3—C2	−2.7 (8)	C28—C27—C31A—O8A	−173.1 (10)
C6—N2—C3—C4	177.5 (5)	C28—C29—C30—C25	2.0 (7)
C6—N3—C9—C8	−4.8 (8)	C28—C29—C32—C33	−142.4 (5)
C6—N3—C9—C10	174.5 (5)	C28—C29—C32—C34	99.6 (6)
C7—N4—C11—C10	0.0 (8)	C28—C29—C32—C35	−21.4 (7)
C7—C8—C9—N3	177.3 (5)	C30—C25—C26—C27	1.2 (7)
C7—C8—C9—C10	−2.0 (7)	C30—C29—C32—C33	38.0 (7)
C8—C9—C10—C11	2.3 (7)	C30—C29—C32—C34	−80.0 (6)
C9—N3—C6—O9	6.6 (9)	C30—C29—C32—C35	159.1 (5)
C9—N3—C6—N2	−174.2 (5)	C31—C27—C28—C29	−176.1 (7)
C9—C10—C11—N4	−1.3 (8)	C31A—C27—C28—C29	174.5 (8)
C11—N4—C7—C8	0.3 (8)	C32—C29—C30—C25	−178.5 (5)

Symmetry codes: (i) *x*−1, *y*, *z*; (ii) *x*+1, *y*, *z*; (iii) −*x*+1, −*y*+1, −*z*.

### Hydrogen-bond geometry (Å, º)

**Table:**

*D*—H···*A*	*D*—H	H···*A*	*D*···*A*	*D*—H···*A*
O10—H10*A*···O2*W*	0.89	1.88	2.658 (7)	144
O10—H10*B*···O1*W*	0.90	1.98	2.768 (7)	146
N3—H3···O4^iv^	0.88	1.92	2.761 (5)	160
C2—H2*A*···O9	0.95	2.23	2.840 (6)	121
C8—H8···O9	0.95	2.34	2.921 (7)	119
C11—H11···O2^iii^	0.95	2.44	2.936 (6)	113
C26—H26···O8*A*	0.95	2.39	2.770 (9)	103
O1*W*—H1*WA*···O1*W*^v^	0.87	2.34	2.918 (10)	124
O2*W*—H2*WA*···O7^vi^	0.87	1.92	2.708 (11)	149
O2*W*—H2*WB*···O3*W*^vi^	0.87	2.38	2.934 (10)	122
O3*W*—H3*WA*···O1^v^	0.87	2.10	2.941 (7)	164
O3*W*—H3*WB*···O2*W*	0.87	2.11	2.912 (11)	154

Symmetry codes: (iii) −*x*+1, −*y*+1, −*z*; (iv) *x*+1, *y*+1, *z*; (v) −*x*+1, −*y*, −*z*+1; (vi) −*x*+2, −*y*, −*z*+1.
